# Supplementary material for: HIVprotI: an integrated web based platform for prediction and design of HIV proteins inhibitors
Source: J Cheminform. 2018 Mar 9;10:12. doi: 10.1186/s13321-018-0266-y (PMC5845081; doi:10.1186/s13321-018-0266-y)
Supplement: Supplementary file 2 — Additional file 2. Source code of HIVProtI web server. [file 13321_2018_266_MOESM2_ESM.zip › HIVprotI_Source-code/hivprotI/ic50/ic50_showmain_draw_new.php]

HIVprotI


php include("head.php");?

php
$seq=$\_POST['sim'];
$email = $\_POST["email"];
$ic = $\_POST["ic"];
if($ic==''){$ic=0;}
$filename = basename($\_FILES['uploadedfile']['name']);
$filena = trim("$filename");
$ran=rand(100,10000);
########### form values ########
#$General = $\_REQUEST['General'];
$int = $\_REQUEST['int'];
$pr = $\_REQUEST['pr'];
$rt = $\_REQUEST['rt'];
#$hiv = $\_REQUEST['hiv'];
#############################
//echo $ran."<br/";
$dir="/home/gpsr/webserver/cgidocs/tmp/manojk/hivproti/first\_$ran";
exec("mkdir $dir");
exec("chmod 777 $dir");
exec("chmod 777 $dir/\*");
if($filena !=''){
move\_uploaded\_file($\_FILES["uploadedfile"]["tmp\_name"],"$dir/input.mol");
}
$FP1=fopen("$dir/input.mol","w");
fwrite($FP1,$seq);
fclose($FP1);
shell\_exec("/usr/bin/babel -i mol $dir/input.mol -o sdf $dir/input.sdf");
############ model selection ###########
$FP2=fopen("$dir/model\_req","w");
#if($General != ""){fwrite($FP2,"$General,");}
if($int != ""){fwrite($FP2,"$int,");}
if($pr != ""){fwrite($FP2,"$pr,");}
if($rt != ""){fwrite($FP2,"$rt,");}
#if($hiv != ""){fwrite($FP2,"$hiv,");}
#if($hupt3 != ""){fwrite($FP2,"$hupt3,");}if($hupt4 != ""){fwrite($FP2,"$hupt4,");}if($kp4 != ""){fwrite($FP2,"$kp4,");}if($mia != ""){fwrite($FP2,"$mia,");}if($mz1 != ""){fwrite($FP2,"$mz1,");}if($panc3 != ""){fwrite($FP2,"$panc3,");}if($panc8 != ""){fwrite($FP2,"$panc8,");}if($panc10 != ""){fwrite($FP2,"$panc10,");}if($psn1 != ""){fwrite($FP2,"$psn1,");}if($sw != ""){fwrite($FP2,"$sw,");}if($yapc != ""){fwrite($FP2,"$yapc,");}
fclose($FP2);
shell\_exec("perl -pi -e 's/,$//g' $dir/model\_req");
shell\_exec("perl -pi -e 's/,/\n/g' $dir/model\_req");
//$models=file\_get\_contents("$dir/model\_req");
//echo"$models  
";
######## main prog########
#shell\_exec("php sub\_draw.php $ran >/dev/null &");
######## main prog########
exec("cp $dir/input.sdf $dir/input.kd");
shell\_exec("/usr/bin/obminimize -ff MMFF94s $dir/input.sdf > $dir/input.pdb");
shell\_exec("/usr/bin/babel -i pdb $dir/input.pdb -o sdf $dir/input\_min.sdf");
shell\_exec("java -jar /home/gpsr/webserver/cgidocs/manojk/hivproti/padel/PaDEL-Descriptor.jar -2d -3d -fingerprints -descriptortypes /home/gpsr/webserver/cgidocs/manojk/hivproti/descriptors.xml -dir $dir/input\_min.sdf -file $dir/desc >/dev/null 2>&1");
#shell\_exec("java -jar /usr1/webserver/cgidocs/servers/avcpred/pd2/PaDEL-Descriptor.jar -descriptortypes /usr1/webserver/cgidocs/servers/avcpred/descriptors1.xml -dir $dir/input\_min.sdf -file $dir/desc -fingerprints -removesalt >/dev/null 2>&1");
exec("perl -pi -e 's/,,/,0,/g' $dir/desc");
exec("perl -pi -e 's/,,/,0,/g' $dir/desc");
exec("perl /home/gpsr/webserver/cgidocs/manojk/hivproti/for\_sever.pl $ran");
shell\_exec("perl /home/gpsr/webserver/cgidocs/manojk/hivproti/knn2\_model.pl /home/gpsr/webserver/cgidocs/manojk/hivproti/arff/list2\_arff $ran");
exec("more $dir/\*/\*\_acc >$dir/all\_acc");
exec("perl /home/gpsr/webserver/cgidocs/manojk/hivproti/parse\_out.pl $ran $dir/all\_acc");
exec("perl /home/gpsr/webserver/cgidocs/manojk/hivproti/parse\_out\_graph.pl $ran");
$line=file\_get\_contents("$dir/out4",'r');
$xaxis=file\_get\_contents("$dir/out44",'r');
$yaxis=file\_get\_contents("$dir/out22",'r');
exec("perl -pi -e 's/\, /\n/g' $dir/out44");
exec("perl -pi -e 's/\, /\n/g' $dir/out22");
exec("paste $dir/model\_req $dir/out22 > $dir/out\_tsort");
$sort = file("$dir/out\_tsort");
##############################
##############################
if($email==""){
echo "";}
else{
echo "";
}
?>

php include "foot.php"; ?
